# Insights into Ergosterol Peroxide’s Trypanocidal Activity

**DOI:** 10.3390/biom9090484

**Published:** 2019-09-12

**Authors:** Thuluz Meza-Menchaca, Angel Ramos-Ligonio, Aracely López-Monteon, Abraham Vidal Limón, Leonid A. Kaluzhskiy, Tatjana V. Shkel, Natallia V. Strushkevich, Luis Felipe Jiménez-García, Lourdes Teresa Agredano Moreno, Verónica Gallegos-García, Jorge Suárez-Medellín, Ángel Trigos

**Affiliations:** 1 Laboratorio de Genómica Humana, Facultad de Medicina, Universidad Veracruzana, Médicos y Odontólogos S/N, Col. Unidad del Bosque, Xalapa C.P. 91010, Veracruz, Mexico; thuluz@gmail.com; 2 LADISER, Inmunología y Biología Molecular, Facultad de Ciencias Químicas, Universidad Veracruzana, Orizaba 94340, Veracruz, Mexico; angramos@uv.mx (A.R.-L.); aralopez@uv.mx (A.L.-M.); 3 Centro de Nanociencias y Nanotecnología, Universidad Nacional Autónoma de México, Carr. Tijuana-Ensenada, Col. Pedregal Playitas, Ensenada C.P. 22860, Baja California, Mexico; avidlimn@gmail.com; 4 Institute of Biomedical Chemistry, 10 building 8, Pogodinskaya Street, 119121 Moscow, Russia; la-kaluzhskiy@yandex.ru; 5 Institute of Bioorganic Chemistry NASB, Kuprevich Street, 220141 Minsk, Belarus; tvshkel@gmail.com (T.V.S.); natstrush@gmail.com (N.V.S.); 6 Laboratorio de Microscopía Electrónica, Facultad de Ciencias, Universidad Nacional Autónoma de México (UNAM), Circuito Exterior, Ciudad Universitaria, México D.F. 04510, Mexico; luisfelipe_jimenez@ciencias.unam.mx; 7 Laboratorio de Nano-Biología Celular, Departamento de Biología Celular, Facultad de Ciencias, Universidad Nacional Autónoma de México (UNAM), Circuito Exterior, Ciudad Universitaria, México D.F. 04510, Mexico; agredano-moreno@ciencias.unam.mx; 8 Facultad de Enfermería y Nutrición, UASLP, Av. Niño Artillero 130, Zona Universitaria Poniente, San Luis Potosí C.P. 78240, Mexico; veronica.gallegos@uaslp.mx; 9 Centro de Investigaciones Cerebrales, Universidad Veracruzana, Xalapa 91190, Mexico; josuarez@uv.mx; 10 Centro de Investigación de Micología Aplicada, Universidad Veracruzana, Xalapa 91010, Veracruz, Mexico

**Keywords:** ergosterol peroxide, endoperoxide, *Trypanosoma cruzi*, SPR, docking, Chagas disease

## Abstract

*Trypanosoma cruzi*, which causes Chagas disease, is a significant health threat in many countries and affects millions of people. Given the magnitude of this disease, a broader understanding of trypanocidal mechanisms is needed to prevent and treat infection. Natural endoperoxides, such as ergosterol peroxide, have been shown to be toxic to parasites without causing harm to human cells or tissues. Although prior studies have demonstrated the trypanocidal activity of ergosterol peroxide, the cellular and molecular mechanisms remain unknown. The results of this study indicate that a free-radical reaction occurs in *T. cruzi* following ergosterol peroxide exposure, leading to cell death. Using a combination of biochemical, microscopic and in silico experimental approaches, we have identified, for the first time, the cellular and molecular cytotoxic mechanism of an ergosterol peroxide obtained from *Pleurotus ostreatus* (Jacq) P. Kumm. f. sp. Florida.

## 1. Introduction

*Trypanosoma cruzi* (Tc) is a hemoflagellate parasite that causes American trypanosomiasis, also known as Chagas disease [1]. Although Chagas disease has afflicted human beings for more than 9000 years B.P. [2], it remains a major health threat, affecting more than five million people worldwide [3]. Another 40 million are at risk for contracting the disease [4,5]. Tc is primarily found in endemic areas of 21 Latin American countries, causing devastating socioeconomic difficulties. The World Health Organization has designated Chagas disease a leading parasitic illness; according to the disability-adjusted life year model, it is one of the most underestimated parasitic malignancies in terms of the numbers of infections, deaths and effects. The disease burden of Chagas disease is five times that of malaria and twenty percent more than HIV. It is also considered the most underestimated human parasite [6]. Every year, 14,000 Chagas disease-related deaths are documented, most of these caused by chronic complications with increasing mortality rates [7,8].

The clinical manifestations of Chagas disease include chronic cardiomyopathy and digestive or neurological alterations that require specialized medical treatment. The infection often occurs when one of several species from the genus *Triatoma* bite a human. After ingesting the human’s blood, the insect defecates on the skin, and the protozoa passes from the insect’s infected feces to the bite site. Infection can also occur through protozoa contact with mucus membranes or by the ingestion of contaminated food. In addition, Tc may be spread during blood transfusions or organ transplants [9]. In a small number of cases, congenital infection can happen at birth through infected mothers [10].

To address the health threat posed by this parasitic disease, two well-known trypanocidal drugs are currently used in clinical practice: nifurtimox and benznidazole [5,11]. These drugs act through the generation of free radicals that are released when they are metabolized. Both drugs have demonstrated side effects, including mutagenic, teratogenic, carcinogenic and reproductive effects [12]. The distribution of these drugs is limited, and they are only available in some countries. Additionally, they do not eradicate the parasite in short-term therapies or during the chronic and latent phases of the disease [11]. Few studies have explored the toxic effects of the metabolites produced by these drugs, particularly those related to benznidazole [13]. A second generation of antimycotic azoles, such as posaconazole, has been developed. These drugs have proven to be more effective, but they have been associated with adverse liver abnormalities [14,15]. While a wide variety of natural products have been shown to display antimicrobial activity [16], trypanocidal potency has not been demonstrated. Chagas disease is considered a challenging illness to treat, and it is difficult to achieve parasite eradication.

Several studies have demonstrated the antiparasitic efficacy of compounds that have been isolated from plants and mushrooms. Endoperoxides, such as artemisinin and ergosterol peroxide, have anti-oncogenic and anti-proliferative properties [17]. Ergosterol peroxide (5α,8α-epidioxy-22E-ergosta-6,22-dien-3β-ol) is an abundant natural compound in many species, including algae [18], plants [19], lichen [20], anemones [21], corals [22], trees [23] 1992) and many fungi [24,25,26,27,28,29,30]. It also has a broad range of biological functions; it is anti-sclerotic [31], pro-apoptotic [32], anti-inflammatory [33], anti-viral [30], anti-mycobacterial [34], anti-proliferative [24], chemo-preventive [35] and, notably, trypanocidal [26]. Recently, the antiamoebic effects of ergosterol peroxide have been reported [36]. Despite this broad range of functions, ergosterol peroxide appears to act selectively. Although many cytotoxic substances exist in nature, only a small group is also innocuous to human cells and tissues. Previously, our research group demonstrated that ergosterol peroxide has a strong effect against Tc [26]. However, there is little experimental evidence related to the compound’s mechanism of action for eradicating Tc. In this report, we describe the trypanocidal activity of ergosterol peroxide, isolated from *Pleurotus ostreatus*, through a mechanism that involves the target CYP51 protein necessary for triggering a free radical reaction that perturb cell organelles.

## 2. Materials and Methods 

### 2.1. Protein Structure Preparation

The crystallographic structures for computational docking in this work are listed in Table 1 unless marked with an asterisk. All structures were downloaded from the Protein Data Bank and, prior to docking, missing side chains and hydrogens were added using the Protein Preparation Wizard routine [37] inside Maestro v.10 (Schrödinger, NY, USA), while ionisable groups were predicted using *propKa* algorithm at pH 7 [38]. All crystallographic structures were minimized and its geometry optimized under the Desmond Molecular Dynamics System version 4.4, (D. E. Shaw Research, NY, USA; Maestro-Desmond Interoperability Tools, version 4.4, Schrödinger, NY, USA.) using the OPLS_2005 force field parameters. Geometry optimization of ergosterol peroxide was performed using a Jaguar software package (Jaguar, version 8.8, Schrödinger, LLC, New York, NY, 2015) with a DFT hybrid method at B3LYP/631-G* level of theory. All minimized structures were read into an AutoDock Tools Framework v1.5.7rc1 and directly prepared to pdbqt input files. All statistical analyses were performed within R statistical software (R core team 2016 v. 3.3.0, R Foundation for Statistical Computing, Vienna, Austria).

### 2.2. Inverse Docking

Inverse docking assays were performed using Autodock v.4.2 [39] and Autodock VINA [40] on a set of crystallographic structures of selected proteins from Tc. Grid size was adjusted to 0.35 Å. The ergosterol peroxide molecule was centred into a ligand binding site for each enzyme. The size and centre of the grid box was set to a cubic size volume of 25 Å, fully covering ergosterol peroxide dimensions. *Gasteiger* charges were added and non-polar hydrogens merged. In addition, 27 crystalline protein structures and 3 homologous models from Tc were selected for docking experiments using Autodock Vina within Autodock 4.2 program software. All docking experiments were carried out in triplicate and Ki was estimated.

### 2.3. Preliminary Surface Plasmon Resonance Experimental Procedures

cDNA for protein expression was isolated using a “DNA-VK” kit of reagents for DNA isolation developed at the Institute of Bioorganic Chemistry of the National Academy of Sciences of Belarus. The surface plasmon resonance (SPR) study of intermolecular interactions was performed using the optical biosensor Biacore 3000 (GE Healthcare, Chicago, IL, USA), operating on a surface plasmon resonance effect. The SPR signal was recorded independently in each channel of the biosensor in resonance units (RU, 1 RU corresponds to 1 pg of protein on the surface of the optical chip) as sensorgrams showing its changing in time. All experiments were performed at 25 °C using standard optical chip CM5 coated with the layer of carboxymethylated dextran. *Kd* values were calculated from sensorgrams sets using software BIAevaluation v.4.1 (GE Healthcare, Chicago, IL, USA). Refractive indexes of compound samples and solvent contained run buffer were measured using a precision refractometer RX-5000 (Atago Co., Ltd., Tokyo, Japan). The following reagents were obtained from manufacturer: HBS-N buffer (150 mM NaCl, 10 mM HEPES, pH 7.4), 1-ethyl-3-(3-dimethylaminopropyl) carbodiimide-HCl (EDC), *N*-hydroxysuccinimide (NHS), 10 mM acetate buffer (pH 4.5), Ni-NTA-agarose (Qiagen, Germantown, MD, USA), Bacto-Tryptone, Bacto-Peptone and Bacto-Yeast extract (DifcoLaboratories, Franklin Lakes, NJ, USA), and Bio-Gel HTP (Bio-Rad, Hercules, CA, USA). Other analytical grade reagents were obtained from local suppliers.

### 2.4. CYP51 Protein Expression Trypanosoma cruzi and Candida albicans

For the CYP51 Tc expression vector inserted into a pCWori vector [41] between NdeI/HindIII restriction sites for transforming an *Escherichia coli* strain HMS174(DE3) carrying the purified and isolated fragment in 12% PAAG gel, Tc cDNA fragments. Previously, the cDNA fragment was produced using GGGGTTGCCTATGCTGCC and CCCCAACGGATACGACGG, as the former forward and latter reverse PCR primers. The PCR amplification was done by using the following conditions: for 5′ at 94 °C, annealing for 1′ at 60 °C, followed extension for 1.5′ at 72 °C, for 30 cycles, and as extension for 10′ at 72 °C as previously described [42]. CYP51 protein expression and purification was performed as follows. A flask of a liter of Broth medium with 1 mM thiamine, 100 mg/mL ampicillin cells were cultured. The broth and cells were incubated in 240 rpm agitation until OD590 reached 1.0 at 37 °C. By adding isopropyl-b-D-thiogalactopyranoside (0.20 mM) and d-aminolevulinic acid, a precursor of heme biosynthesis (final concentration 0.5 mM) the protein CYP51 was induced. After 48 h, the cells were harvested and lysed for protein purification. For *C. albicans*, cDNA samples were isolated from a patient exhibiting resistance to antifungal drugs, and were used for obtaining CYP51. Four amino acid substitutions (K-179-E, L-224-I, G-307-C, M-372-T) were found in the case of an *C. albicans* amino acid sequence [43]. In both cases for *C. albicans* and *T. cruzi*, the immobilization of CYP51 on the surface of the optical chip CM5. HBS-N buffer was used as a running buffer. Carboxyl groups of dextran were activated by passing the mixture of equal volumes of 0.2 M EDC and 0.05 M NHS for 7 min at a flow rate 5 µL/min followed by washing with a running buffer at the same speed for 1 min. Immobilization of CYP51 was performed by passing the protein solution (20 µg/mL) in 10 mM of acetate buffer (pH 4.5) for 10 min at a flow rate of 5 µL/min. After that, the chip surface was washed with running buffer for 1 h at a flow rate 5 µL/min. The effectiveness of protein immobilization was on average 10,000 RU.

### 2.5. Mushroom Strain

A strain CP-26 of *Pleurotus ostreatus* (Jacq.) P. Kumm. f. sp. Florida was employed. Fruiting bodies were cultivated in barley straw as described in a previous report [44].

### 2.6. General Procedures

Mushroom samples from *Pleurotus ostreatus* were taxonomically identified and dehydrated in an oven at 60 °C with airflow for 30 h. Distilled solvents were used. Chromatographic columns were dry packed with silica gel (230–400 mesh, Merck, USA), and glass plates of silica gel (Merck 60 GF254 of 0.2 mm thickness) were used for thin-layer chromatography. Iodine vapors were used to crate spots. The melting point was determined by a Fisher–Johns apparatus and was not corrected. ^+^H experiments were performed using a Varian Gemini 2000 apparatus Palo Alto, California (See Supplementary Information Figure S1).

### 2.7. Isolation and Purification of Ergosterol Peroxide

Fractions were eluted with hexane: EtOAc (7:3) of the chromatographic purification of the ethyl acetate extract from the 3.367 kg of dry fruiting body of *Pleurotus ostreatus*. In addition, 130 mg of a white crystalline compound (m.p. 179–181 °C) were obtained and identified as ergosterol peroxide based on its NMR spectroscopic data and compared to an authentic sample same as previously described [36].

### 2.8. Parasite Cultures

The strain used for the present study was the epimastigotes form of Tc Y in all experiments isolated from vector *Triatoma infestans*. A liver infusion tryptone broth-LIT supplemented with 10% fetal bovine serum was used as a growing media [45].

### 2.9. Carbonylation Activity in Extracellular Epimastigotes

The Tc culture strain Y started with a cell density of 1 × 10^6^ on a 50 mL volume until reaching the log phase epimastigotes measured by direct counting with a haemocytometer by mL. The cells were washed with sterile Phosphate-buffered saline (PBS) twice, each time centrifuging at 2000× *g* over 10′. The pellet was dissolved in an LIT complete media until reaching a density of 4 × 10^5^ parasites per ml. An amount of 500 µL of cellular suspension was added to a 96-well plate and incubated until analysis. The parasites were incubated for 24 h at 28 °C and also treated with ergosterol peroxide (40 µM) that was previously dissolved in less than 1% dimethyl sulphoxide (DMSO). A negative control was added with H_2_O_2_, and another with no treatment other than ultrapure MQ H_2_O and a third one with a mix of ultrapure MQ H_2_O and Dodecyl Bisulfate DMSO in a concentration of less than 1%. All conditions were performed in triplicates and repeated three times independently and then to processed by a statistical analysis. For the carbonylation analyses, *Trypanosomes* were lysed and the quantification of carbonyl groups were determined. After treatment, a single ml mixture of reaction blended with 1 ml of 20 mM of 2,4-dinitrophenylhydrazine in 2 M HCl, which forms a stable dinitrophenyl product that is then read spectrophotometrically. The solution was incubated for at 25 °C and subsequently a 15% trichloroacetic acid was added to the tube. Plates were kept on an ice bucket for 10 min to right after spin down of the cells at 12,000× *g* for 20 min. The remaining protein pellet was kept and washed twice with an ethanol/ethyl acetate (1:1 *v*/*v*) solution and dissolved in 1.5 mL of 8 M guanidine-HCL (pH 7.4) and finally vortexed for suspension. The solubilized hydrazones were measured at 370 nm. The concentration of carbonyl content was counted by using the molar absorption coefficient 22,000 M^−1^ cm^−1^ D. Hartley, [46]. The concentration of 2,4-dinitrophenylhydrazine DNPH derivatized proteins was determined by the molar extinction.

### 2.10. Electron Microscopy Assay

Tc samples were treated with 10 µM ergosterol peroxide for 12 h were fixed to be analyzed using a JEOL JSM-7600F scanning electron microscope (Wilmington, Massachusetts, USA) at a working distance of 15 to 21 mm and a voltage of 1.2 to 5.0 kV after a preliminary wash in distilled water, followed by dehydration in a series of ethanol solutions of increasing concentration (30%, 50%, 70%, 90% and 100%), air drying on 0.5″ aluminum mounts and sputter coating with gold-palladium using a Polaron SC7640 High Resolution Sputter Coater (Quorum Technologies, Newhaven, East Sussex, UK); some micrographs were obtained using a Philips XL-30 environmental SEM (Pl. 7 and 8). Line drawings were made from digital images.

### 2.11. Confocal Imaging

Tc samples were treated with 20 µM ergosterol peroxide for 24 h were fixed to be analyzed. Images of confocal microscopy were obtained by using a Leica SP8 laser scanning confocal microscope (Leica, Wetzlar, Germany), equipped with a HC PL APO CS2 63x/1.40 oil immersion lens (Leica, Wetzlar, Germany), at 50% argon laser. A 545 nm Helium–Neon laser line was used for excitation of propidium iodide with a 645 nm emission filter.

### 2.12. Transmission Electron Microscopy

Tc samples were dosed with 20 µM ergosterol peroxide and after 24 h were fixed to be analyzed. Pellets were processed for standard electron microscopy [47]. Briefly, fragments were fixed 4 h at room temperature in a mixture of 2.5% glutaraldehyde and 4% paraformaldehyde, buffered in PBS (pH 7.2). Post-fixation was performed with 1% osmic acid two hours. Samples were subsequently dehydrated in a graded series of ethanol and embedded in an epoxy resin at 60 °C for 48 h. A contrast was conducted with 5% uranyl acetate and 0.5% lead citrate. Grids were observed with a transmission electron microscope (JEOL 1010, JEOL, Peabody, MA, USA) working at 80 kV. Images were obtained with a charge-coupled device camera coupled to the microscope. 

### 2.13. Candida albicans Viability Assay

The following microbial strains were used: *C. albicans* CAF2-1 [48], which was derived from *C. albicans* SC5314 (ATCC MYA2876), *C. glabrata* (ATCC 2001) and *S. cerevisiae* BY4741. Yeast cells were cultivated overnight in 250 mL flasks in 50 mL of yeast extract–peptone–dextrose, YPD medium at 30 °C. A preculture was prepared by diluting the overnight culture to an optical density OD620 of 0.2 in 25 mL of YPD medium, and the yeast cells were allowed to grow for 3 h so that they reached the exponential growth phase. The OD was determined in a 180-ll sample volume with the microtiter plate reader lQuant (BioTek Instruments, Bad Friedrichshall, Germany). The working culture was prepared by diluting the preculture to an OD620 of 0.2. After cultivation for another 3 h, the OD620 was recorded, the whole suspension was taken into a Falcon tube, and cells were harvested by centrifugation (Eppendorf centrifuge 5804 R) at room temperature at 5000 rpm for 5 min and washed carefully three times with phosphate-buffered saline (PBS). The washed cells were resuspended in 1 mL of PBS. To test the electrochemical activity of nonliving organisms (dead cells), the working culture was autoclaved at 121 °C for 20 min. The cells were harvested into lysogeny broth, LB broth media and resuspended in a 96-well plate in a concentration of with 10^5^ CFU/mL to be treated with the ergosterol peroxide using the final concentrations of 1, 5, 10, 20, 40, 70 µM. The *C. albicans* yeast cells were grown at 30 °C for 3, 18, 24 and 48 h and were harvested, resuspended to be counted in a Neubauer camera MilliporeSigma (St. Louis, MI, USA) for final estimation of concentration and statistical analysis.

### 2.14. Analysis of Intermolecular SPR Interactions

Stock solutions of the compound were prepared by dissolving it in DMSO at a concentration of 5 mM. These stock solutions were further diluted by HBS-N buffer up to a concentration of 100 µM. To obtain solutions with lower concentrations of compounds, HBS-N buffer with the addition of DMSO in the same concentration as that of 100 µM solution was used. To prevent bulk-effects during SPR analysis DMSO contained, HBS-N buffer was used as a run buffer. The alignment of DMSO concentration in HBS-N buffer and compound samples was performed by value of refractive index. To determine the equilibrium dissociation constant of compound complex with immobilized CYP51A1 (*Kd*), sets of sensorgrams were obtained by injection of compound solutions for 6 min at a flow rate of 30 µL/min in the range of 10–75 µM concentrations. Dissociation of the complexes was recorded for at least 30 min at a flow rate 30 µL/min. After that, the sensor chip surface was regenerated by injections of the regenerating solution (2 M NaCl, 0.4% m/v CHAPS) for 17 s at a flow rate 35 µL/min.

## 3. Results

### 3.1. Ergosterol Peroxide Interacts with Tc Sterols and Does Not Target Tc Glucose Metabolism

**Figure 1:**
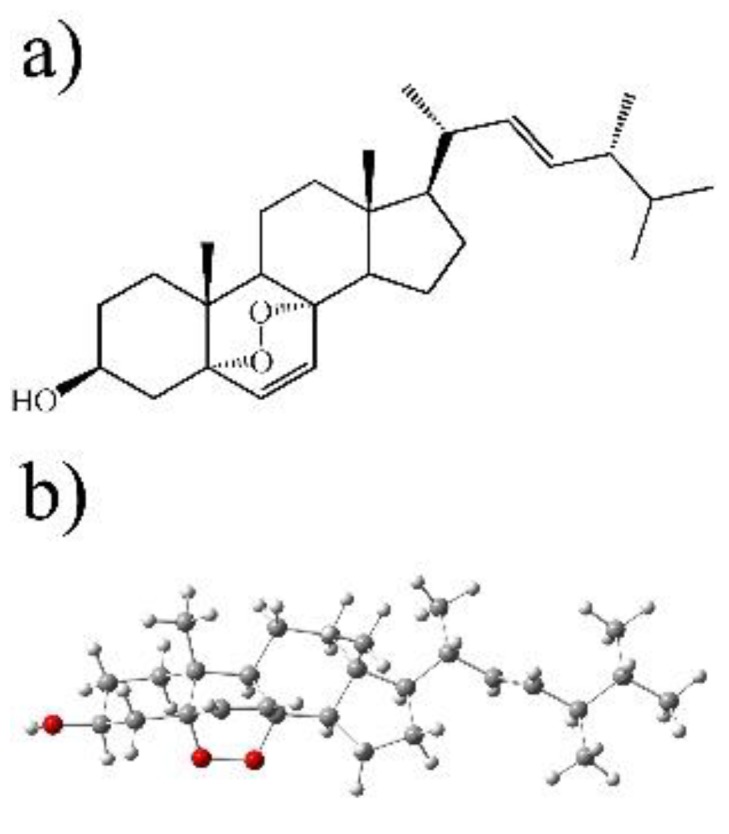
Molecular structure of ergosterol peroxide. (**a**) contains a side carbon 2D-chain structure, and (**b**) image contains a 3D-carbon-chain structure. The oxygen components are highlighted in red.

**Figure 2:**
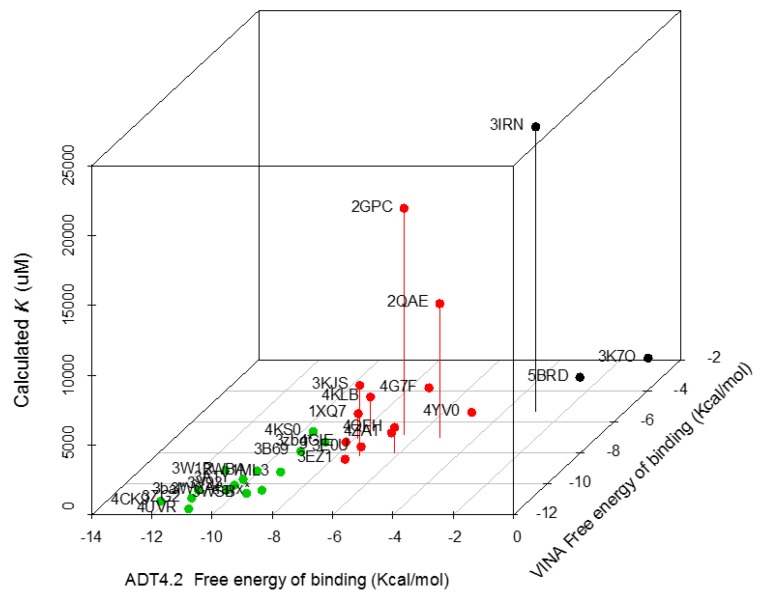
The clustering of free energy of binding values versus the calculated ∆G values for ergosterol peroxide docked to the *T. cruzi* protein dataset. The hierarchical clustering plot was derived from two docking calculations of the *T. cruzi* protein dataset against ergosterol peroxide. The first subset of *T. cruzi* proteins (green dots) displayed the lowest free energies of binding (−14 to −10 Kcal/mol) in both methods, resulting in the lowest calculated ∆G. The second subset (red dots) had higher free energies of binding (<8 Kcal/mol) than the first subset. The third subset (black dots) had the highest free energies of binding for interaction with ergosterol peroxide.

Ergosterol peroxide has a similar structure to ergosterol and is likely ingested by Tc cells (Figure 1). To identify potential Tc proteins that might directly interact with ergosterol peroxide, an inverse-docking screening was performed on a subset of Tc protein crystallography structures. The relative binding affinities of ergosterol peroxide were assessed against this Tc protein library. We selected 30 of the 280 Tc protein structures in the Protein Data Bank, filtering out redundant and partial-length peptides. The proteins were selected based on X-ray resolution (2.5–1.5 Å), and the binding free energy of ergosterol peroxide was calculated for each protein using two methods. The binding free-energy expressed in −∆G (Kcal/mol) value was calculated using AutoDock version 4.2 (Table 1). The proteins with the lowest predicted ∆G values shared specific binding sites for proteins that were involved in glycolysis (glucose-6-phosphate isomerase and glucokinase), redox mediation (old yellow enzyme, dihydrofolate reductase, lipoamide dehydrogenase and prostaglandin F synthase), reduced peptide-binding cleft pockets (cruzain, cyclophilin and spermidine synthase) and various catalytic mechanisms proteins (Fe-SOD, lipoamide dehydrogenase, pyruvate kinase and tyrosine phosphatase). The list included several known SPR, including glutathione peroxidase, cruzain, sterol 14-alpha demethylase, trans-sialidase, dihydrofolate reductase, trypanothione reductase, glyceraldehyde 3-phosphate dehydrogenase, old yellow enzyme and phosphodiesterase. The lowest ∆G were associated with proteins related to membrane transport, such as ABC transporters, and lipid metabolism, including C-8 sterol demethylase, prostaglandin F synthase, farnesyl-transferase, sterol carrier protein 2, sterol 14-alpha demethylase (CYP51), squalene synthase and lanosterol 14-alpha demethylase (Figure 2). The latter of these were likely highlighted due to their hydrophobic cores. A comparison of posaconazole and ergosterol peroxide binding modes to CYP51 P450 of T. *cruzi* proteins was performed (See Figure S2).

### 3.2. CYP51 Interacts with Ergosterol Peroxide in Tc but Not in Candida albicans

**Figure 3:**
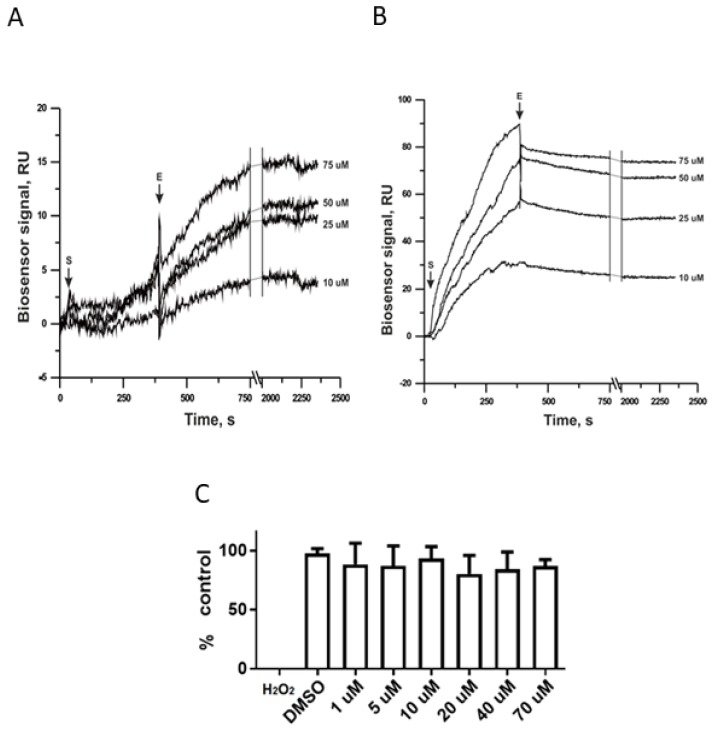
Sensograms of the surface plasmon resonance SPR assays and survival assays. (**A**) SPR from *Candida* ssp. and (**B**) SPR from Tc, both showing a correlation between sensor signal and time per second at a range of affinity concentration subsets (10 µM, 25 µM, 50 µM and 75 µM). The data are representative of the results of three independent experiments. Both (**A**,**B**) show association and disassociation effect **E** of interaction in the arrow at the top; (**C**) as negative control results of a *C. albicans* survival test in the presence of ergosterol peroxide through a concentration gradient, taken from the average of three time windows (18, 24 and 48 h). Each bar represents the results of three independent experiments.

We selected CYP51 as the Tc protein with the top predictive value from our molecular docking model. Then, we developed an SPR assay to compare the ergosterol peroxide interactions with the Tc (B) and *C. albicans* (A) homologous of this protein. As shown below, there is a concordance at the effect in association and disassociation and ergosterol peroxide concentration and resonance with the Tc version of CYP51; there was no correlation effect with the *C. albicans* counterpart (Figure 3). Interestingly, ergosterol peroxide survival assays of *C. albicans* and Tc showed no observable effect in the former and a significant effect on the latter, with an IC_50_ = 6.74 µg/mL [30].

### 3.3. Detection of Protein Carbonylation Assay

**Figure 4:**
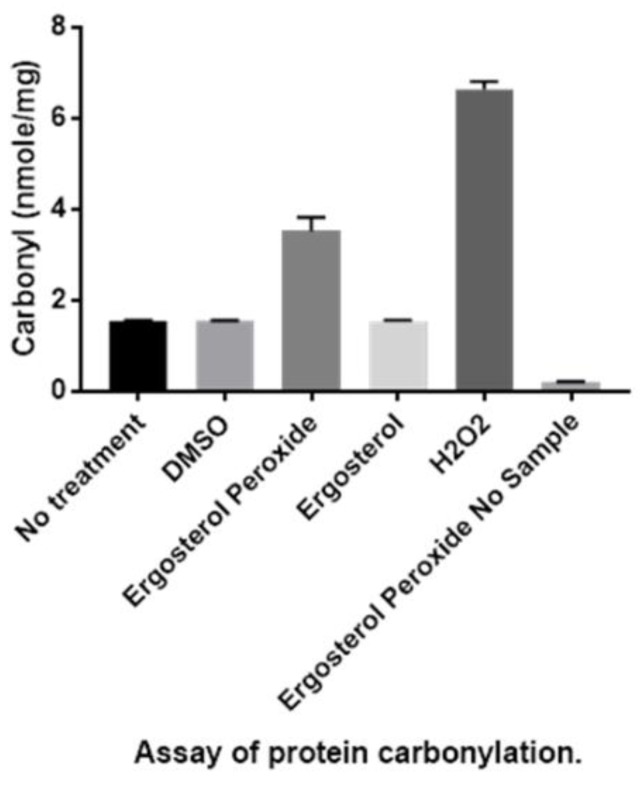
Graphic bar displaying the carbonyl concentrations of *T. cruzi* hemoflagellates after various treatments. Lane 1, no treatment; lane 2 (less than 1% dimethyl sulfoxide); lane 3, ergosterol peroxide (40 µM); lane 4, ergosterol (40 µM); lane 5, hydrogen peroxide (120 µM) and lane 6, ergosterol peroxide, no hemoflagellate sample. In all conditions described 10^4^ of cells per ml were treated. Ergosterol peroxide treatment was significant with a *p* ≤ 0.05 tested with non-parametric one-way ANOVA.

To examine the ergosterol peroxide activity from a biochemical perspective, we tested its cytotoxicity on the Tc hemoflagellate. These results suggest that the compound’s trypanocidal activity is due to the breakage of its oxygen-oxygen bond, unleashing oxidation onto lipids, proteins and nucleic acids. To determine if this free radical damage was caused by the rupture of the peroxide bridge, we measured the post-treatment carboxyl output using a carbonylation assay (Figure 4). Confocal and transmission electron microscopy were used to observe the ergosterol peroxide reactivity on the carbonylated proteins of Tc.

Significant concentrations of carbonylated compounds were observed in the Tc hemoflagellate samples that were treated with ergosterol peroxide compared with those that were treated with ergosterol or the vehicle (dimethyl sulfoxide) and compared to the samples that received no treatments. The detection of carbonylated proteins after ergosterol peroxide treatment suggests that the trypanocidal activity is related to the peroxide bridge, whose homolytic cleavage triggers oxidation.

### 3.4. Detection of Enlarged Extracellular Vesicle Structures after Ergosterol Peroxide Treatment

**Figure 5:**
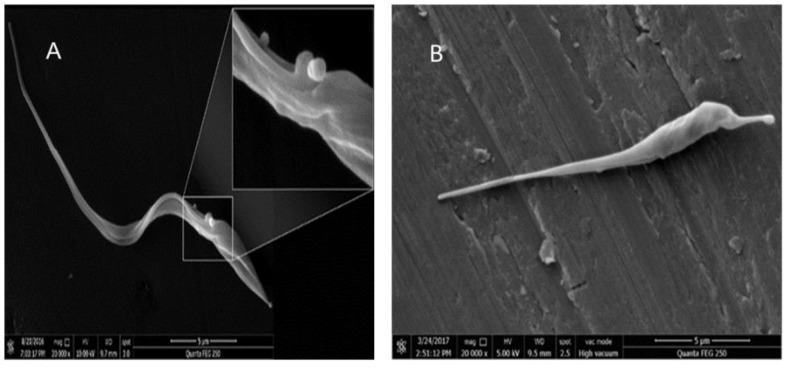
Scanning electron microscopy of the *T. cruzi* hemoflagellate specimens treated with ergosterol peroxide. (**A**) the left panel is shown at 20,000× magnification, and the top right panel is an image of the same sample but magnified; (**B**) the untreated negative control at 20,000× magnification.

To examine the evidence of cellular membrane disruptions, we treated Tc hemoflagellate specimens with a cytotoxic concentration of ergosterol peroxide (10 µM) and incubated the specimens for 12 h. Enlarged extracellular vesicles were observed in all the analyzed specimens that were treated with ergosterol peroxide compared to the non-treated specimens (Figure 5A). The significantly larger vesicles (on average) were likely due to the cytotoxicity of the endoperoxide compound (Figure 5B).

### 3.5. Ergosterol Peroxide Disruption of the Cytoplasmic and Nuclear Membranes

Similar experimental conditions were used with a different microscopy approach to evaluate cell viability and to identify the active site of the ergosterol peroxide within the Tc cells. The ergosterol peroxide was applied for a longer period, and confocal electron microscopy was used to observe the Tc cells. The Tc samples were treated with a 20 µM cytotoxic concentration of ergosterol peroxide, stained with propidium iodine, incubated for 24 h and then analyzed with confocal electron microscopy. The fluorescence was detected by probing the in situ interactions between the ergosterol peroxide and the Tc nucleic acids, the result of nuclear and cytoplasmic membrane disruptions caused by the ergosterol peroxide.

**Figure 6:**
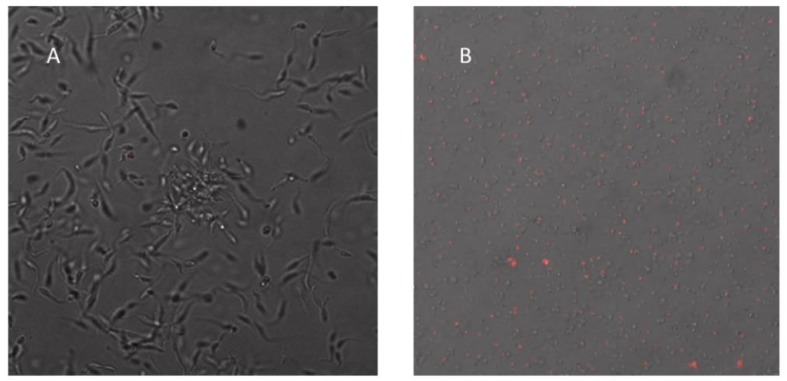
Nuclear and cytoplasmic membrane disruption of T. *cruzi* after ergosterol peroxide treatment. The confocal microscopy images are shown at 1000× (**A**) the control, untreated *T. cruzi*; (**B**) *T. cruzi* treated with 20 µM ergosterol peroxide for 24 h.

These interactions were made evident by the positive signal produced by the binding of the propidium iodine and the Tc nucleic acids (Figure 6A,B).

### 3.6. Ergosterol Peroxide Disruption of the Golgi Apparatus and the Cytoplasmic and Nuclear Membranes

**Figure 7:**
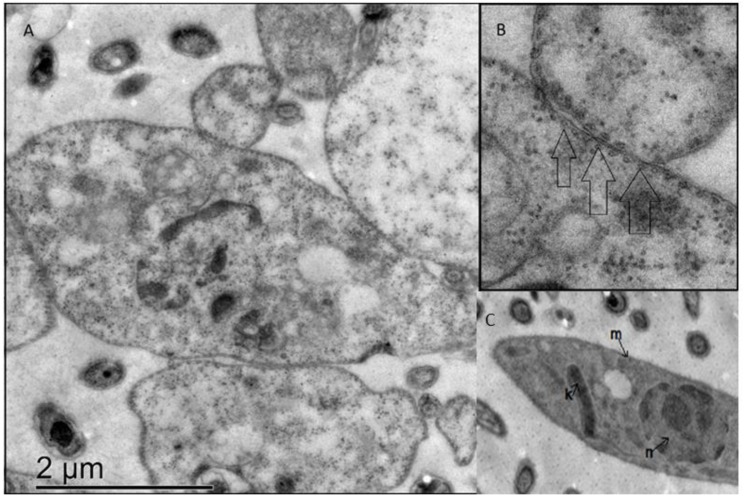
Transmission electronic microscopy images of a *T. cruzi* sample treated with 20 µM of ergosterol peroxide for 24 h. (**A**) treated sample, left panoramic, right amplification; (**B**) higher with arrows showing pores; (**C**) untreated sample showing (n) nucleus (m) membrane (k) kinetoplast.

After a longer ergosterol peroxide exposure, we observed changes in the Tc nuclear and cytoplasmic membranes. In addition, the Golgi apparatus disappeared (Figure 7).

## 4. Discussion

The biological effects of ergosterol peroxide (5α,8α-epidioxy-22E-ergosta-6,22-dien-3β-ol) range from immunosuppressive to cytotoxic to anti-microbial and include anti-plasmodial, anti-leishmanial, anti-amoebic and trypanocidal activities [29,36,49,50]. However, the mechanism of action remains unknown. This study focused on ergosterol peroxide’s antitrypanosomal function. Reverse docking was used to identify Tc proteins that were capable of interacting with ergosterol peroxide. A broad set of Tc crystallographic protein structures were reviewed to detect any informative patterns and to explain previously reported observations. We believe that there is a toxicity with ergosterol, but this is significantly lower than EP [51]. Our aim was not to estimate levels of toxicity in ergosterol and ergosterol peroxide. However, by comparing both compounds, we ascertained that the actual difference is the oxygen/oxygen bound at the peroxide bridge that enables interaction with the heme-binding site of CYP51. In a consecutive step, this can trigger free radicals though homolytic cleavage at the O-O bound, the pharmacophore responsible for its biological activity.

The key identified proteins included enzymes that are involved in sugar and lipid metabolism, but the highest docking predictions were for enzymes that are indispensable in sterol metabolism, rather than glucose metabolism. The top match that was predicted in silico to have molecular interactions with ergosterol peroxide was CYP51, a sterol demethylase and lipid transporter. This protein has been recently proved to be essential for *Trypanosoma brucei* [52]. CYP51 contains two different domains; in one, it interacts with the NADPH and there is another one that contains the heme group. Our hypothesis, due to docking results, is that it does not have an effect as EP binds to a different domain; due to this, it may not have any interaction with the NADPH cofactor. It can affect indirectly in case there is damage with the protein but is not expected to have any direct effect.

The molecular docking results for CYP51 were confirmed with SPR, which indicated a significant interaction with the Tc version of CYP51 and no evidence of affinity with the *C. albicans* version of CYP51. These results are consistent with our previous studies that have shown ergosterol peroxide cytotoxicity in Tc but not in *C. albicans.* The Candida genus is capable of synthesizing sterols, so these organisms do not depend on sterol intake from the environment. Tc may also synthesize its own sterols, as it is able to grow in potato-dextrose agar broth, which lacks ergosterol. The resistance to EP by *Candida albicans* might be due to differences in CYP51 aminoacid sequence.

However, specific sterols are required to maintain Tc viability and proliferative capacity during the parasite’s infection and life cycle. Sterols are also indispensable for several membrane-related cellular functions, including the integration of hydrophobic domains, membrane fluidity and permeability, and the formation of ion channels, enzymes and cellular compartments [53,54]. CYP51 has been shown to interact with the ergosterol of fungi, yeasts, plants and other microorganisms, such as Tc. Our electron scanning microcopy images showed enlarged extracellular vesicles, indicating that ergosterol peroxide treatment affected the membrane permeability of our Tc samples.

The structural similarity of ergosterol peroxide and ergosterol explains both our initial in silico results as well as the cytotoxic effect of ergosterol peroxide on Tc. The structural resemblance of cholesterol, ergosterol and ergosterol peroxide further explains the ability of ergosterol peroxide to incorporate into the parasite’s plasma membranes.

The reverse docking results indicated that ergosterol peroxide had a high affinity with transport enzymes and sterol synthesis proteins (CYP51). This might explain how ergosterol peroxide is dispersed across the cytoplasm. Enzymes involved in transport and sterol synthesis were identified as having a high probability of interacting with ergosterol peroxide. Consequently, once ergosterol peroxide enters a cell, it may disrupt metabolic output and lipid-mediated transport by interacting with CYP51, an important sterol synthesis enzyme. Notably, CYP51 is a primary drug target for several microbial infections in both animals and plants; its potential pharmacological role should not be overlooked.

The transmission electron microscope images revealed that the predominant changes following ergosterol peroxide treatment occurred at the nuclear, kynetoplasm and cytoplasmic membranes. The cytoplasmic membrane, in particular, exhibited lineal and equidistant pores. After ergosterol peroxide treatment, the nucleic acid content was disseminated throughout the cytoplasm, as shown by the propidium iodide staining.

These results were further confirmed by the carbonylation test, which demonstrated an increase in carbonyl groups after ergosterol peroxide treatment. These carbonyl groups are generated by free radical reactions, which can be produced through the homolytic cleavage of peroxide bonds and which may be enhanced by the transition metals present in Tc. The ergosterol peroxide mechanism of action may involve a reactive oxygen species that causes the membrane alterations we observed.

Biological systems are constantly exposed to reactive oxidants that are either the result of endogenous production or are externally delivered (i.e., from drugs, photo-oxidation or pollutants). These reactive oxidants cause molecular transformations and oxidative damage. Inside cells, proteins are the primary targets for oxidative damage due to their abundance and naturally-engineered reaction rates, but the high reactivity of oxidants means their detrimental effects can be traced to all molecular components, including proteins, lipids, DNA, RNA, carbohydrates and even antioxidants [55]. Oxidative damage promotes protein degradation, uncouples electron transport, impairs respiration, unpairs transcription and sensitizes cellular membranes through lipid peroxidation and the formation of reactive aldehydes. Organic endoperoxides are potential generators of highly reactive oxygen species that can disrupt lipids and proteins with the production of alkoxy radicals. The first step in this mechanism likely involves an endoperoxide reaction with a transition metal that promotes the homolytic cleavage of a peroxide bond and the release of a highly reactive alkoxy radical, which reacts in situ.

Previous studies have reported that iron catalyzes the formation of alkoxy radicals from hydroperoxides of cholesterol through the Fenton reaction. Based on the results of the present study and the close similarity between ergosterol, cholesterol and ergosterol peroxide, we propose that a Fenton mechanism is likely involved in the homolytic cleavage of the peroxide bridge. Since the active site of CYP51 contains a heme iron, a radical burst could be triggered by its interaction with ergosterol peroxide, which would act as an enzymatic inhibitor generating long-lived radicals or carbonyl moieties to name a few examples [56]. The function of these radicals as enzyme inhibitors, or as generators of secondary free radicals and metabolite inactivators, could enhance their chemotherapeutic effects against Tc [24,57]. With these two mechanisms taking place separately or in conjunction, Tc might enter apoptosis. The blebbings we observed in the confocal microscopy images might be formed through a caspase cascade, as occurs in other eukaryotes [58,59,60]. As a transporter enzyme, an ergosterol peroxide-activated CYP51 protein could be disseminated throughout the cytoplasm and come into contact with other CYP51 proteins, which are also found in the external part of the cell nucleus [61]. Consequently, ergosterol presence may be affected, impairing cellular, nuclear and mitochondrial/cytoplasmic membranes. The above may cause membrane alterations, such as blebbings. Thus, it is not strange that CYP51 has been promoted as a candidate as anti-leishmanial or anti-trypanosomal drug [62]. Previous reports have demonstrated that particular changes to cell morphology occur during apoptosis, including membrane contraction and the formation of membrane-enclosed extracellular vesicles called blebbings. The actin–myosin system has been implicated in the contractile repercussion forces that lead to the formation of blebs, although the biochemical and transcriptional pathways that drive this apoptosis contraction have not been elucidated. The virulence factors that cause blebbings are well-characterized and include bacteria, protozoa, helminths and fungi [63,64,65]. In our study, the transmission electron microscopy showed that the cytoplasmic membrane alterations were paralleled by the disruption of the Golgi apparatus and the nuclear membrane. The confocal microscopy also showed that the nuclear membrane was affected and revealed that ergosterol peroxide could have reached the nucleic acids. These results correlate with previous findings [66], which reported in vitro evidence that ergosterol peroxide may inhibit the beta polymerase. However, our docking prediction value for beta polymerase indicated a low binding probability (data not shown).

## 5. Conclusions

**Figure 8:**
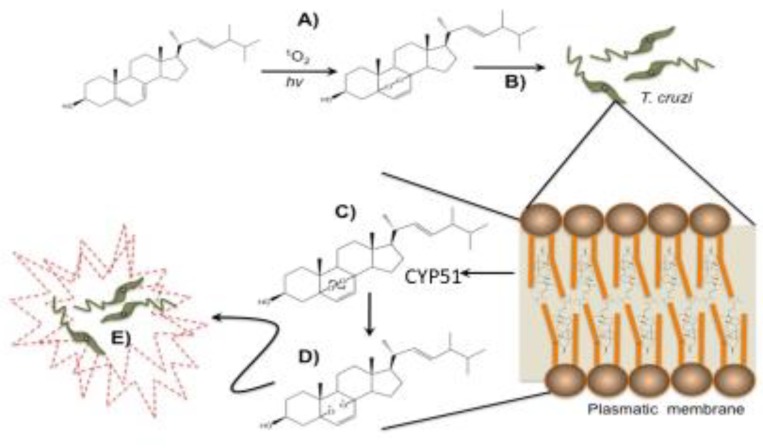
A hypothetical mechanism of action for the ergosterol peroxide oxidative burst. (**A**) ergosterol peroxide oxidation reaction; (**B**) ergosterol peroxide membrane docking; (**C**,**D**) breakage of the covalent oxygen bound after CYP51 association and triggering of the free radical reaction; (**E**) Tc cytotoxicity.

Our results indicate that a specific lipid/protein oxidation is involved in ergosterol peroxide’s interaction with CYP51. Ergosterol peroxide’s high affinity for interacting proteins allows it to deliver a deathly cargo. This mechanism links alkoxy radical formation to the production of lipid aldehydes and protein carbonylation and explains the rapid (within 24 h) cytotoxic effect of ergosterol peroxide on Tc. Ergosterol peroxide promotes proteomic and lipid carbonylation, providing evidence of the development of free radicals and a specific directed mechanism. In the Tc cell membrane, when ergosterol is replaced by ergosterol peroxide, the peroxide moiety is broken, triggering a series of free radical reactions (Figure 8). The alkoxy radicals are capable of reacting with the fatty acids, among others, of the membrane phospholipids or with triacylglycerols, generating aldehydes. However, more research should also be conducted to confirm the effects of ergosterol peroxide on the transcriptional machinery and nuclear and cytoplasmic membranes of Tc. Finally, the dramatic changes to the cellular membranes after ergosterol peroxide treatment suggest an indirect lipid oxidation through radical reactions.

## Figures and Tables

**Table 1:** The best matches between T. *cruzi* proteins and ergosterol peroxide in descending order of ∆G predictive value.

*Trypanosoma* spp. Protein Set	PDB Code	VINA Free energy of Binding (Kcal/moL)	ADT4.2 Free energy of Binding (Kcal/moL)	Calculated *Ki* (µM)
Sterol 14-alpha demethylase (CYP51)	4CK9	−11.1	−12.21	0.002
Lanosterol 14 alpha demethylase	3ZG2	−10.9	−11.29	0.013
Farnesyl transferase	3WSB	−10.6	−9.64	0.087
C-8 sterol demethylase model	3bal *	−10.4	−11.35	4.537
Squalene synthase (TcSQS)	3WCA	−10.4	−10.42	0.021
ABC b model	4ayx *	−10.4	−9.24	0.171
Phosphodiesterase	3V93	−10.1	−10.32	28.66
Old Yellow enzyme (FMN oxidereductase)	3ATY	−9.7	−10.25	0.029
Glyceraldehyde 3-phosphate dehydrogenase	1ML3	−9.4	−9.17	191.147
Trypanothione reductase	2WBA	−9.2	−10.06	44.723
Dihydroorotate dehydrogenase	3W1R	−9.1	−11.17	6.407
Chagasin	3EZ1	−8.4	−7.59	2.9
Dihydrofolate reductase	3KJS	−8.2	−7.21	5060
Gluc-6-Pho Isomerase	4QFH	−8	−6.17	1830
Trans-sialidase	3B69	−7.9	−9.34	0.145
Glutathione peroxidase	3E0U	−7.6	−7.5	3.24
Prostaglandin F synthase	4GIE	−7.3	−8.17	1.507
Sterol carrier protein 2 model	3zbg *	−7.3	−8.86	0.321
Cyclophilin	1XQ7	−7.1	−7.88	1810
Lipoamide dehydrogenase	2QAE	−7	−5.22	9576.667
Cruzain	4KLB	−6.9	−7.58	2783.333
Fe-Superoxide dismutase	2GPC	−6.8	−6.52	16,176.667
Pyruvate kinase	4KS0	−6.7	−9.59	91.953
Spermidine synthase	4YV0	−5.6	−4.94	241.627
Dihydrofolate Reductase	3IRN	−5.3	−2.98	2,0330
Enolase	4G7F	−3.9	−7.3	102.843
Glucokinase	5BRD	−3.4	−2.57	310.043
Ribose 5-phosphate isomerase	3K7O	−2.1	−1.03	238.85

* Models fetched by existing heterologous structures.

## Supplementary Materials

The following are available online at https://www.mdpi.com/2218-273X/9/9/484/s1, Figures S1 and S2.
