# Supplementary material for: Insights into Ergosterol Peroxide’s Trypanocidal Activity
Source: Biomolecules. 2019 Sep 12;9(9):484. doi: 10.3390/biom9090484 (PMC6770379; doi:10.3390/biom9090484)
Supplement: Supplementary file 1 [file biomolecules-09-00484-s001.pdf]

SUPPEMENTARY INFORMATION

A)

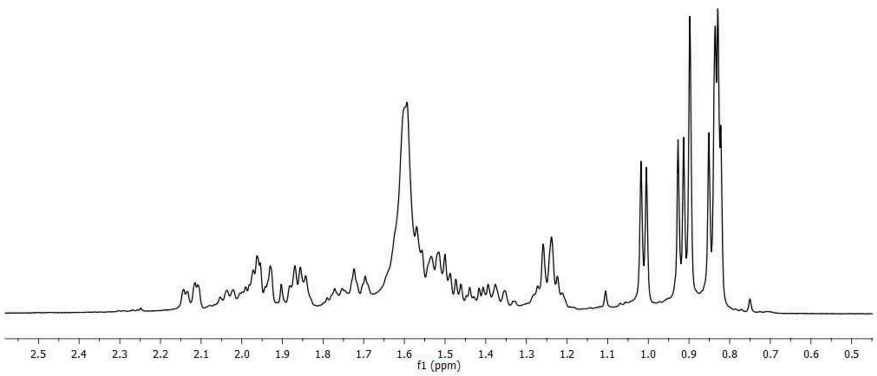

B)

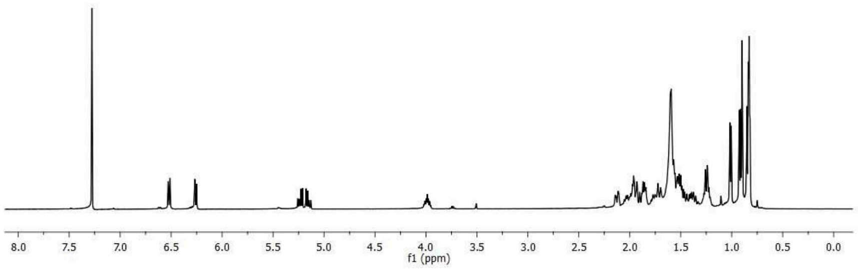

SI-1. Proton NMR spectrum for ergosterol peroxide (5α,8α-epidioxy-22E-ergosta-6,22-dien-3β-ol). In image A amplified spectrum and B compete signal spectrum.

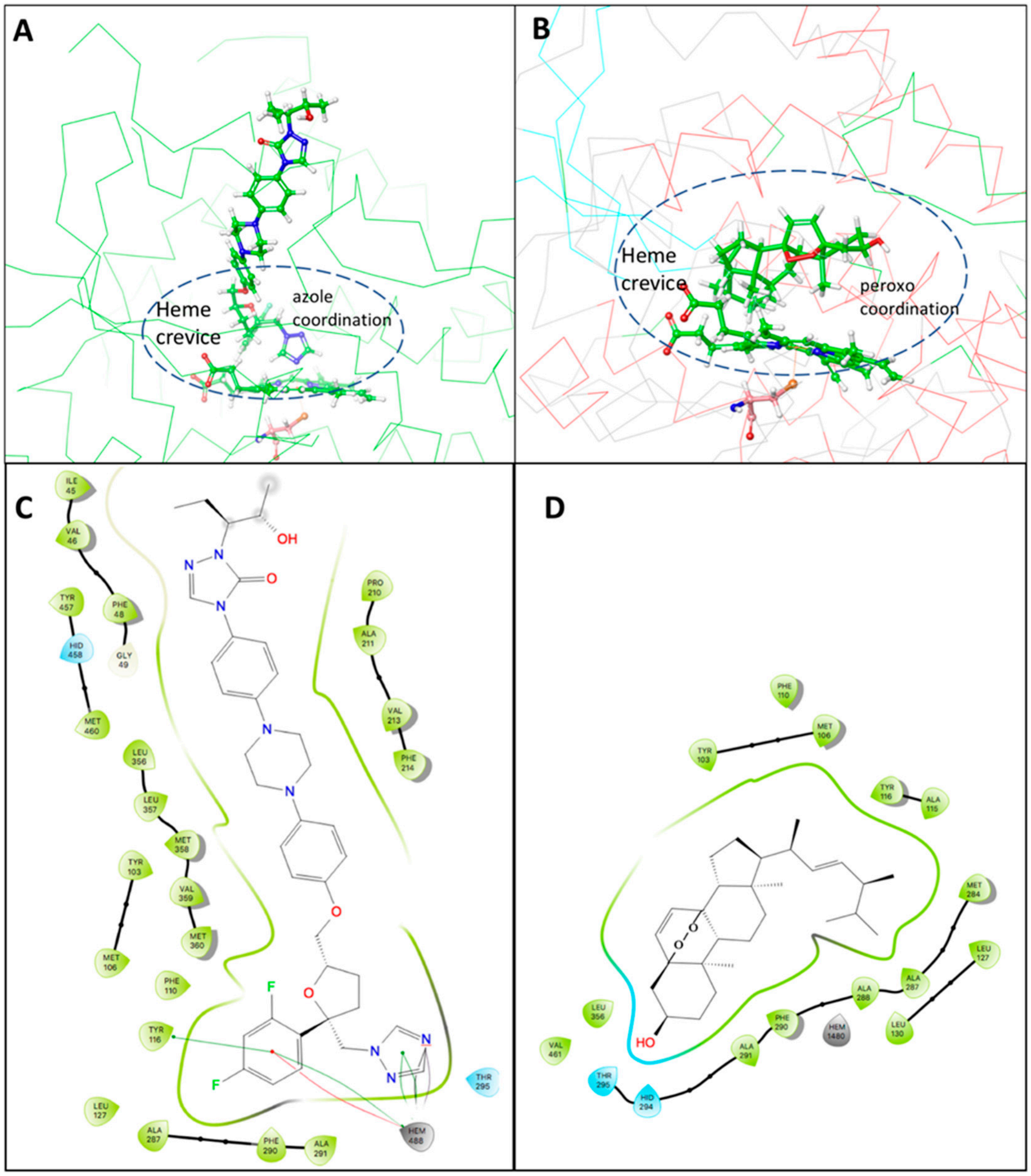

SI-2 Comparison of posaconazole and ergosterol peroxide binding modes to CYP51 P450 of T. cruzi proteins: A) co-crystallized posaconazole complexed with CYP51 P450 (PDB code: 3K1O); B) ergosterol peroxide complexed with CYP51 P450 of T. cruzi. The binding site between both ligands is the same (next to the heme group, in its crevice); C) ligand interaction diagram of the whole interaction zone between posaconazole and CPY51 P450; D) ligand interaction diagram of the proposed interaction zone between ergosterol peroxide and CYP51 P450.
